# Social Media Engagement and Usage Patterns, Mental Health Comorbidities, and Empathic Measures in an Italian Adolescent Sample: A Comparative Study

**DOI:** 10.3390/children12091226

**Published:** 2025-09-13

**Authors:** Ilaria Accorinti, Giulia Mutti, Pamela Fantozzi, Annarita Milone, Gianluca Sesso, Greta Tolomei, Elena Valente, Antonio Narzisi, Edoardo Martinelli, Maria Rosaria Cordella, Gabriele Masi, Stefano Berloffa

**Affiliations:** 1 IRCCS Fondazione Stella Maris, Viale del Tirreno 331, Calambrone, 56128 Pisa, Italy; 2 Department of Clinical and Experimental Medicine, University of Pisa, 56126 Pisa, Italy; 3 IMT School for Advanced Studies, 19 Piazza San Francesco, 55100 Lucca, Italy

**Keywords:** social media addiction risk, empathic abilities, social media category, adolescent mental health

## Abstract

Background: The link between problematic social media (SM) use and socio-emotional deficits has limited clinical evidence. This study compares SM addiction risk and empathic abilities between psychiatric outpatients and healthy peers, exploring how SM categories and/or diagnostic category may modulate these relations. Methods: A total of 362 Italian adolescents (11–18 years; 147 cases, 215 controls) completed the Social-Media Disorder Scale (SOMEDIS), Bergen Social-Media Addiction Scale (BSMAS), Interpersonal Reactivity Index (IRI), and Reading the Mind in the Eyes test (RME). Self-reported daily online time and most used social media platforms were recorded. Results: Clinical participants showed higher mean SOMEDIS (M = 18.37) and BSMAS scores (M = 11.71) compared with controls (both *p* < 0.001). Females reported longer daily SM use (χ^2^ = 5.4, *p* = 0.020). Positive associations were observed between SM addiction risk and age. Within the clinical group, adolescents with emotional dysregulation displayed higher problematic use scores; a modest correlation emerged with internalizing symptoms (withdrawn—depression). Regarding empathy, higher SM addiction risk correlated with lower cognitive empathy (IRI Perspective Taking, Fantasy) and higher Personal Distress. Platform type showed small differences: users of “Profiling” platforms reported lower empathy scores compared to “Entertainment” users. Conclusions: Adolescents with psychiatric conditions appear more vulnerable to problematic SM use and reduced empathic abilities. Associations were modest, and platform effects were limited. These findings should be considered exploratory; larger longitudinal studies are needed to clarify causal pathways between SM use, empathy, and adolescent mental health.

## 1. Introduction

Social media (SM) encompasses user-generated platforms that allow people to share information and content on the internet, especially on social network sites (e.g., TikTok, YouTube, Instagram, Facebook, Snapchat) [1].

The technological advancements in recent years, the proliferation of ‘digital natives’ (i.e., children and adolescents raised with digital technology), and the global coronavirus pandemic have all contributed to a marked increase in the frequency and duration of social media (SM) usage. It has been hypothesized that individuals may attempt to compensate for a lack of face-to-face interaction by incorporating social media into their lives as a matter of course [2]. In 2024, the global population of social media users surpassed five billion, with an average daily engagement time of approximately 151 min [3]. Evidence indicates that preferences for different SM platforms vary according to age: adolescents and emerging adults predominantly use YouTube, Instagram and TikTok, whose short-form, visually driven, and interactive formats have been shown to confer feelings of strong reward and immediate gratification. The pervasiveness of smartphone access facilitates the permeation of SM activity into most routine contexts, thereby lowering the threshold at which intensive use may begin to disrupt sleep, academic or occupational performance, and face-to-face relationships [4].

According to the latest WHO survey conducted in 2021/2022 on adolescent social media use and gaming in Europe, central Asia and Canada, 36% of adolescents reported continuous online contact with others [5]. In the instance of such interference, the behavioral pattern manifests addiction-like characteristics and is associated with adverse outcomes in mental health, educational attainment, and community functioning. Despite the fact that “social media addiction” is not currently recognized as a diagnostic entity in the Diagnostic Statistical Manual of Mental Disorders—5th Edition (DSM-5), the scientific literature has adopted the construct of problematic social media use (PSMU) [6] to capture clinically relevant patterns of over-consumption. Research work in this domain is increasing, with the aim of refining operational criteria, while psychometric instruments (such as the Bergen Social Media Addiction Scale (BSMAS) [7]) are already available.

Empathy is a complex multidimensional concept which forms the basis of social cognition and is composed of affective empathy (AE), the ability of sharing others’ feelings, and cognitive empathy (CE), the ability of understanding others’ emotions [8,9]. Empathy is an innate ability and humans start to be aware of emotions very early in life. During childhood and adolescence, cognitive empathy and prosocial behavior gradually develop and improve along a non-linear path, due to several individual and environmental variables [10]. Theory of Mind is a distinct domain of social cognition that enables individuals to make inferences regarding others’ mental states. This capacity, coupled with the ability to recognize emotions and empathize, facilitates the development of successful social interaction [11].

The ubiquity of the internet and social media brought profound changes in our everyday life, including our understanding of social interaction and cognition. As an example, the concept of digital and virtual empathy is emerging and seems to have a role in online communication, including emotional and supportive expressions [12,13].

According to the latest author knowledge, evidence linking patterns of social media use, empathic abilities and mental health conditions is inconclusive.

In particular, the link between social media use and empathy is still not well established or understood: while certain studies suggest no significant correlations between these two constructs [14,15,16,17], some authors have demonstrated the opposite [18,19,20]. Another hypothesis is that this relationship could be mediated by other factors, such as alexithymia [18].

Concurrently, the relationship between social media and mental health is complex and multi-faceted, with some studies highlighting the positive aspects, such as self-expression, communication, and entertainment, while other studies focused more on adverse effects, such as cyberbullying, addictive behaviors, and the spread of misinformation [21,22]. Overall, difficulties in emotion regulation can be associated with higher risk of problematic social media use [23], and a recent systematic review identified a significant association between social media use and adolescent depression, without a clear causality [24]. Moreover, the last study by Dwijayanti and Pratiwi found a significant negative relationship between social media addiction and students’ mental health [25].

Finally, the relationship between empathy and psychiatric disorders is also not straightforward: while affective empathy might be linked to increased vulnerability to depression (especially during adolescence) [26], cognitive empathy is often impaired in conditions such as schizophrenia and bipolar disorder, with differential effects across disorders and components of empathy [27,28].

Taken together, the current literature highlights three main gaps. First, evidence on the relationship between social media use and empathy remains inconsistent, with studies reporting both null [14,15,16,17] and significant associations [18,19,20]. Second, it remains unclear how psychiatric disorders shape this relationship. For instance, affective empathy has been associated with vulnerability to depressive symptoms [26], whereas cognitive empathy is often impaired in schizophrenia and bipolar disorder [27,28]. Moreover, recent studies suggest that specific platforms may relate differently to mental-health disorders, but inconclusive results are available. Use of highly visual platforms has been linked to internalizing symptoms, with body-image concerns acting as a mediator [29], eating-disorder pathology [30] and higher risk of children and adolescents being engaged in non-suicidal self-injury [31]. In young adults, results can be variable, as time spent on TikTok and YouTube was related to poorer mental-health outcomes, whereas Snapchat was linked to lower anxiety and loneliness and higher peer support [32].

This study directly addresses these gaps by comparing adolescents with psychiatric disorders to healthy peers, with the aim of (i) clarifying whether clinical populations exhibit higher SM addiction risk and distinct empathic profiles, (ii) whether diagnostic subgroups differ in these associations, and (iii) whether patterns of SM use modulate the relationship between social media, empathy, and mental health.

## 2. Materials and Methods

### 2.1. Participants

This cross-sectional comparative study comprised 362 participants, aged 11–18 years, divided into clinical and control groups. Recruitment windows were March–December 2024 (clinical) and March 2024–March 2025 (control group). Participants in the clinical sample were consecutively recruited at the Department of Child and Adolescent Psychiatry and Psychopharmacology at the IRCCS Stella Maris Foundation hospital. Controls were recruited in Tuscany and Piedmont via existing school collaborations. This approach was adopted to ensure the following: feasibility and rapid enrolment, broadened geographic heterogeneity beyond the hospital catchment area, and mitigation of recruitment bias from a single locale.

Inclusion was restricted to those aged 11–18 years to balance measurement validity and developmental relevance. First, adolescents in this age range can independently and reliably complete the study questionnaires without parental assistance, minimizing proxy bias and interviewer effects. Second, this is the developmental window in which independent social media use typically emerges and consolidates, ensuring sufficient variability in exposure for meaningful analysis. To address residual age-related heterogeneity, we adjusted all models for age and sex.

This study was conducted in accordance with the Declaration of Helsinki and approved by the Regional Ethics Committee for Clinical Trials of Tuscany (Pediatric Ethics Committee at Meyer Children Hospital of Florence; date of approval: 13 February 2024; protocol number: 12/2024; protocol code: Videoemp24). All participants and their parents were informed about the assessment procedure, and participation in this study was voluntary.

Clinical psychiatric diagnoses were established according to the Diagnostic and Statistical Manual of Mental Disorders—Fifth Edition (DSM-5) [33] based on medical history, clinical observations, and a semi-structured diagnostic interview: the Schedule for Affective Disorders and Schizophrenia for School-Age Children—Present and Lifetime Version (K-SADS-PL [34]). The interview was administered by trained child psychiatrists to both parents and adolescents and subsequently revised by senior child psychiatrists. Therefore, the clinical sample included individuals diagnosed with Mood Disorders, Behavioral Disorders, Attention Deficit Hyperactivity Disorder (ADHD), Autism Spectrum Disorder (ASD), Tic Disorder, Neurodevelopmental Disorder, Anxiety Disorder, and Obsessive–Compulsive Disorder (OCD).

Inclusion criteria were as follows: age range 11–18 years, presence of any psychopathological diagnosis according to DSM-5, and intellectual ability within the normal range of Total Intelligent Quotient (TQI) or General Ability Index (GAI) ≥ 85, evaluated with the Wechsler Intelligence Scale for Children—Fourth Edition (WISC-IV) [35] or the Wechsler Adult Intelligence Scale—Fourth Edition (WAIS-IV) [36]. Patients were excluded in cases of comorbid intellectual disability and/or psychotic condition (given the inability to complete the questionnaires), presence of active epilepsy, neurological conditions and/or neurosensory deficits (i.e., visual and auditory).

### 2.2. Questionnaires

All participants completed the following questionnaires:

- IRI (Interpersonal Reactivity Index [37,38]), a self-report questionnaire that, in its Italian-translated and standardized version, investigates empathic abilities through 28 items and 4 subscales: perspective taking (PT) and fantasy (F) contribute to the cognitive empathy domain (EC); empathic concern (CE) and personal distress (PD) contribute to the affective empathy domain (AE). A total score can be obtained as representative of general empathic abilities of the subject. Scores range from −14 to +14 though no cut-off score was set. The IRI Italian version shows good reliability and validity in the Italian cultural context [34].
- RME (Reading the Mind in the Eyes [10,39,40]) consists of 28 pictures in the ocular facial region of women and men, each accompanied by four words related to a mental or emotional state; the child is asked to choose the one that best represents what the person in the picture is feeling. It is considered an advanced Theory of Mind test where the greatest score represents higher social ability in making inferences regarding others’ mental and emotional states; it is currently available in Italy with good reliability and validity in the Italian cultural context in both its adult and child versions (the latter is the version used in the study) [39].
- SOMEDIS (The Social Media Use Disorder Scale for Adolescents [41]), a self-report questionnaire to assess social media problematic use based on ICD-11 and DSM-5 criteria for gaming disorders. It consists of 10 questions (9 to evaluate SM usage patterns and an extra question to assess frequency and duration of problems). The Italian validation of this questionnaire was conducted by the Italian National Institute of Health [42]. A higher total score (specifically > 20) indicates an increased risk for problematic social media use.
- BSMAS (Bergen Social Media Addiction Scale [43]), a self-report questionnaire which contains six items reflecting core addiction elements (i.e., salience, mood modification, tolerance, withdrawal, conflict, and relapse). In the Italian version, no clinical cut-off figure was officially established at compilation time [44], so the total score of 24 was considered clinical according to the latest national and international methodologies [45].

In addition to validated instruments, a brief set of additional self-report questions, created for the purpose of evaluating social media usage (i.e., most-used platform, estimated daily time) was administered. These items were designed for descriptive purpose only, were not derived from a validated psychometric tool, and employed fixed categories for frequency/time and a checklist for platforms. For analysis, platforms were grouped into four distinct groups based on the predominant rationale for utilization (see Table 1) [46]. In addition, the platforms that are mostly highly visual and utilize short-form content (Instagram and TikTok) were isolated for the purpose of investigating potential differences to other platforms. Frequency of use was categorized as follows: low frequency of use (between 0 and 2 h per day), average frequency of use (between 2 and 4 h per day), and high frequency of use (over 4 h per day) [47].

In the clinical sample, clinical severity was assessed via the Clinical Global Impression-Severity score (CGI-S) [48], and according to the overall adaptive functioning with the Children Global Assessment Scales (C-GAS) [49]. The K-SADS-PL was conducted with parents and adolescents. Psychopathology assessment was completed using the Child Behavior Checklist (CBCL [50]), a 118-item parent-report questionnaire, and the Youth Self Report (YSR), the 113-item self-report version completed by adolescents. Both instruments provide scores across three broad dimensions (total problems, internalizing problems, and externalizing problems), eight syndrome scales, and six scales aligned with the DSM diagnostic categories. Finally, cognitive function was assessed using the Wechsler Intelligence Scale for Children—Fourth Edition (WISC-IV) [35] or Wechsler Adult Intelligence Scale—Fourth Edition (WAIS-IV) [36], according to the age range.

### 2.3. Statistical Analysis

Statistical analyses were conducted using JASP software (version 0.19.3 Apple Silicon). Continuous variables are reported as mean accompanied by standard deviation (SD), while categorical variables are presented as frequency (%). A *p*-value of less than 0.05 (two-tailed) was considered statistically significant.

Significant differences (*p*-value < 0.05) in dichotomic variables between independent groups were assessed using the Pearson χ^2^ test. Univariate comparisons of continuous variables between independent groups were conducted using Student’s *t*-test, which was performed only after normality (Shapiro–Wilk test) and homogeneity of variances (Levene’s test) were confirmed. The nonparametric Mann–Whitney U test was applied when normality could not be established. Analysis of variance (ANOVA) was used after normality assumption was verified through the Shapiro–Wilk test and used to assess significant differences (*p*-value < 0.05) in continuous clinical variables across demographic groups. In cases where the normality was not met, the Kruskal–Wallis rank test was employed. Post hoc comparisons were performed using Tukey’s test whenever ANOVA led to a statistically significant result to identify significant comparisons between variables. Bivariate relations between questionnaire scores were explored with Pearson’s correlations (Spearman when assumptions were violated). Partial correlations controlling for age were computed for all clinically restricted analyses.

## 3. Results

The study sample consisted of 362 participants, comprising 148 females (40.9% [95% CI: 35.8–46.1%]), 211 males (58.3% [95% CI: 53.0–63.4%]), and 3 individuals (0.8% [95% CI: 0.2–2.4%]) who chose not to disclose their gender. Participants’ age ranged from 11 to 18 years, with a mean age of 13.30 years (SD = ±2.04). The sample was composed of 147 individuals (40.6% [95% CI: 35.5–45.9%]) classified as the clinical group, and 215 (59.4% [95% CI: 54.1–64.5%]) as healthy controls. In the clinical group, there were 43 females (29.3% [95% CI: 22–37.3%]), 102 males (69.4% [95% CI: 61.3–76.7%]), and 2 individuals (1.4% [95% CI: 0.2–4.8%]) who did not declare their gender. The mean age in this group was 14.07 years (SD = ±2.46). In the control group, there were 105 females (48.8% [95% CI: 42–55.7%]), 109 males (50.7% [95% CI: 43.8–57.6%]), and 1 individual (0.5% [95% CI: 0–2.6%]) who did not disclose their gender. The mean age in the control group was 12.54 years (SD = ±1.08).

Evaluating the features of social media addiction risk in the two groups, social media use was frequent in both (clinical group = 88.43% [95% CI: 82.1–93.1%], controls = 91.62% [95% CI: 87.1–95.0%]; χ^2^ = 1.019, *p* = 0.31). When analyzed by gender, 94.6% [95% CI: 89.6–97.6%] of females and 87.2% [95% CI: 81.9–91.4%] of males reported using social media. Statistical significance (χ^2^ = 5.4, *p* = 0.020) is observed, suggesting higher social media use among females compared to males.

**Figure 1:**
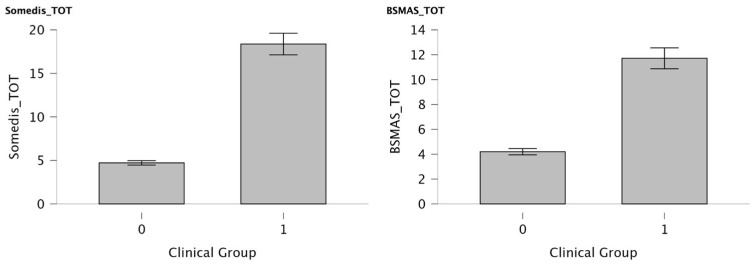
Bar plots showing mean total scores in SOMEDIS and BSMAS questionnaires between the two groups. Group 0 corresponds to healthy controls, and Group 1 to the clinical group. Error bars indicate standard error.

Scores on both the SOMEDIS and BSMAS questionnaires differed according to clinical status. Specifically, participants in the clinical group scored significantly higher than controls on both measures, as reported in Figure 1 (SOMEDIS mean score in clinical group: 18.37; mean score in control group: 4.72; *p* < 0.001; effect size: 0.995; BSMAS mean score in clinical group: 11.71; mean score in control group: 4.2; *p* < 0.001; effect size: 0.916). Notably, none of the participants in the healthy control group exceeded the clinical cut-off on either questionnaire.

No gender-based differences were observed in the questionnaires’ results (SOMEDIS mean score in females: 9.53; mean score in males: 10.73; *p* = 0.168; BSMAS mean score in females: 7.10; mean score in males: 7.34; *p* = 0.841). Self-reported daily time on social media correlated positively with the total scores of both measures (SOMEDIS: *p* < 0.001; BSMAS: *p* < 0.001).

According to age, a direct correlation was observed with SOMEDIS (t = 4.913, *p* < 0.001, effect size 0.470) with higher scores in older participants; this correlation was not observed with the BSMAS total score (*p* = 0.097). A direct correlation was observed between age and social media self-reported frequency use (F = 4.393, *p* = 0.013); post hoc analysis showed a significant difference between the groups with low- and high-frequency use (*p* = 0.031) and low- and average-frequency use (*p* = 0.011).

### 3.1. Clinical Subgroup Analyses

In order to investigate the relationships between psychiatric disorders and social media addiction risk, to evaluate possible correlations between psychopathological features, empathic competences, and social media use, a deeper analysis on the clinical group (*n* = 147 participants) was conducted.

According to the main clinical diagnosis, our sample was composed as follows (Table 2): 23 participants had a main diagnosis of Attention Deficit Hyperactivity Disorder (ADHD), 4 of Autism Spectrum Disorder (ASD), 3 of Tic Disorder, 18 of Neurodevelopmental Disorder, 18 of Anxiety Disorder, 8 of Obsessive–Compulsive Disorder (OCD), 4 of Behavioral Disorder, and 65 of Mood Disorder, and 4 were classified as “other”. A correlation study was conducted, and no relationship emerged.

Dysregulation patterns (emotional, alimentary, and cyclothymia) emerged from an in-depth analysis of the clinical group. As these symptoms are considered transdiagnostic, they were deemed relevant for further investigation into underlying mechanisms. Patients exhibiting dysregulation patterns scored higher scores on both SOMEDIS (*p* = 0.024; effect size 0.409) and BSMAS (*p* = 0.006; effect size 0.498) questionnaires compared to other individuals in the clinical sample.

Finally, independent *t*-Tests were conducted to evaluate possible correlations between a single diagnosis and social media addiction risk; an inverse correlation was observed between tic disorder/obsessive–compulsive disorder and the SOMEDIS questionnaires (*p* = 0.040; effect size: −0.438).

A correlation study was performed to evaluate the relationship between clinical pictures including severity of symptoms (measured using CGI and CGAS scores), psychopathology features (measured by CBCL and YSR questionnaires), cognitive abilities, and social media addiction risk. A direct relationship between the CBCL withdrawn depression subscale and SOMEDIS total scale was observed (*p* = 0.040, Spearman’s rho = 0.188). An inverse correlation was observed between the Verbal Comprehension Index (WISC-IV VCI) and SOMEDIS total score, indicating that participants with lower VCI could rank higher on the SOMEDIS questionnaire (*p* = 0.011, Spearman’s rho = −0.232). Correlations were conducted, regressing out age of participants.

Differences in short-form-content SM use were also explored according to clinical diagnosis. A significant difference was observed for the ASD diagnosis (χ^2^ = 11.66, *p* = 0.003): a greater use of short-form-content SM was found in participants without ASD diagnosis.

### 3.2. Social Media Use and Empathic Measures

**Figure 2:**
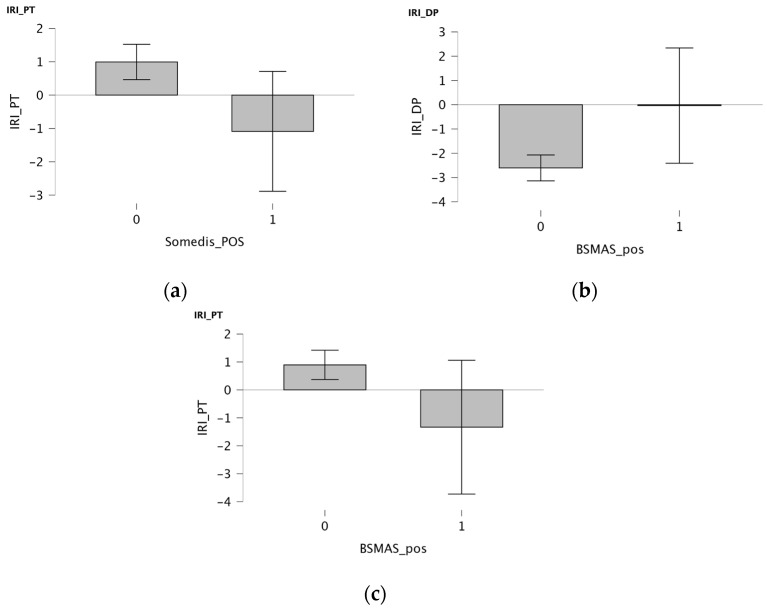
(**a**) Bar plots showing mean total scores on the IRI Perspective Taking subscale, grouped by SOMEDIS questionnaire positivity. (**b**) Bar plots showing mean total scores in IRI Personal Distress subscale, grouped by BSMAS questionnaire positivity. (**c**) Bar plots showing mean total scores in IRI Perspective Taking subscale, grouped by BSMAS questionnaire positivity.

Differences in empathic abilities in relation to SM use were explored using Independent *t*-Tests (Figure 2). A significant negative association was observed between the Perspective Taking IRI subscale (PT) and SOMEDIS (T student = −2.652, *p* = 0.008; effect size = −0.418), suggesting that participants with lower perspective-taking abilities scored higher in SOMEDIS. No significative associations were observed with BSMAS (Italian version) using the cut-off of 24, currently used in international methodologies [41]. Since a clinical cut-off has not been officially set yet for the BSMAS (Italian version), a deeper analysis was conducted using the total score of 17 as the cut-off for a medium-high risk of SM addiction. Consequently, a significant negative association was observed between the Perspective Taking IRI subscale and BSMAS (T student = −2.235, *p* = 0.026; effect size = −0.447), meaning that participants with lower perspective-taking abilities scored higher in BSMAS. A significant direct correlation was found between BSMAS and the Personal Distress IRI subscale (PD) (T student = 2.549, *p* = 0.011; effect size = 0.233) with lower PD scores in participants with a BSMAS score under the clinical cut-off.

Relationships between empathic abilities and SM use were also explored using a correlation study. A weak negative correlation between the IRI Fantasy subscale and SOMEDIS score (*p* = 0.018, Spearman’s rho = −0.125) was observed.

Since none of the healthy controls surpassed either the SOMEDIS or BSMAS cut-off, case–control comparisons were not feasible for these binary variables. Instead, a correlation analysis was conducted on cases and control groups: the former saw a direct correlation between BSMAS and IRI Fantasy subscale (*p* = 0.021, Spearman’s rho = 0.191), IRI Personal Distress subscale (*p* < 0.001, Spearman’s rho = 0.369) and Affective Empathy domain (Spearman’s rho = 0.307, *p* < 0.001). A direct correlation also appeared between SOMEDIS and IRI Fantasy subscale (*p* = 0.046, Spearman’s rho = 0.165), IRI Personal Distress (*p* < 0.001, Spearman’s rho = 0.301), and IRI Affective Empathy (Spearman’s rho = 0.201, *p* = 0.015); an inverse correlation transpired with the IRI Perspective Taking subscale (Spearman’s rho = −0.212, *p* = 0.010). The control group saw a marginal direct correlation between the SOMEDIS score and IRI Affective Empathy domain (*p* = 0.005, Spearman’s rho = 0.1662) and between the BSMAS score and IRI Personal Distress subscale (*p* = 0.028, Spearman’s rho = 0.180). Correlations were conducted regressing out age of participants.

No meaningful correlations emerged for the RME test.

**Figure 3:**
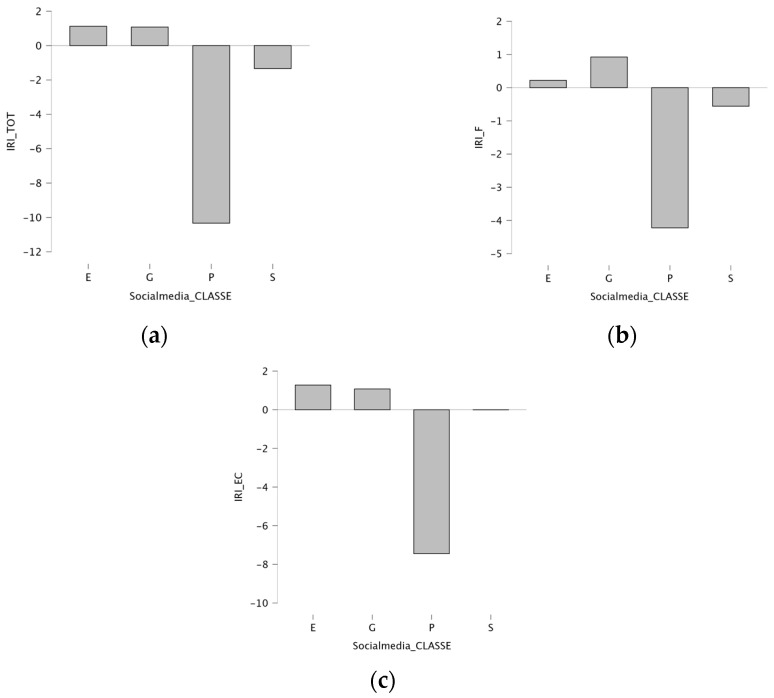
(**a**) Bar plots showing mean total scores on the IRI Total scale, grouped by social media platform. (**b**) Bar plots showing mean total scores in IRI Fantasy subscale, grouped by social media platform. (**c**) Bar plots showing mean total scores in IRI Cognitive Empathy subscale, grouped by social media platform.

Finally, to explore the relationships among psychopathological features, empathic competences, and social media use based on usage patterns, social media platforms were categorized into four groups: Entertainment, Sharing Content, Profiling, and General Purpose. SOMEDIS and BSMAS scores did not differ across categories, nor did the participants’ RME test performance. Conversely, the IRI total score revealed significant differences based on the platform used (F = 3.474, *p* = 0.016, η^2^ = 0.031). Contrasts emerged between the “Entertainment” and “Profiling” categories (*p* = 0.023), with the latter showing lower scores in post hoc testing (Figure 3a). Similar results were observed for the Fantasy IRI subscale (F = 2.808, *p* = 0.040, η^2^ = 0.025), where “Entertainment” scored higher than “Profiling” (*p* = 0.047) (Figure 3b). In the Cognitive Empathy domain (F = 3.528, *p* = 0.015, η^2^ = 0.031), “Profiling” scored lower than both “Sharing Content” (*p* = 0.041) and “Entertainment” (*p* = 0.011) categories (Figure 3c).

## 4. Discussion

The present study aims to expand on and develop the existing research on the association between social media use, empathy, and mental health conditions, with a specific focus on social media usage patterns. This was achieved by comparing a sample of adolescents diagnosed with psychiatric disorders and a sample of healthy controls.

Social media addiction (SMA) is a recent nosographic construct that has yet to be defined and recognized as a diagnostic disorder. However, it has already been observed to present a high risk in the younger population. In particular, adolescents aged 16–24 have shown increased vulnerability to excessive social media [51,52], with the younger population showing higher usage rates [16,53]. Consistent with this trend, epidemiological results from our sample revealed a direct correlation between age and both self-reported SM frequency use and SM addiction risk (as indicated by higher SOMEDIS scores in older participants). This could also reflect the fact that older adolescents generally have greater autonomy and reduced parental monitoring, which translates into higher daily use [54]. At the same time, adolescence is a developmental stage characterized by rapid biological, cognitive, and social changes [55], and empathic abilities themselves undergo significant, non-linear changes during this period [10]. This creates a dynamic scenario in which social media usage patterns vary according to age-related needs and developmental tasks. However, further longitudinal studies are needed to clarify how developmental stage and social media use interact in shaping empathic capacities and mental health outcomes.

Regarding possible gender differences, discrepancies emerged in the self-reported data showing higher SM usage among females, while the questionnaire scores revealed no statistical difference between males and females. This is also consistent with the results reported in the existing literature: while some studies have observed higher SM usage in females, some others suggested males may be prone to greater addiction-risk behaviors [15,56]. Besides inconsistencies in usage frequencies, scholars agree that SM usage patterns differ by gender: in general, while females tend to prefer image-centric, relationship-oriented activities such as chatting and blogging platforms, males spend more time in competitive/gaming environments [57,58]. Gender differences in social comparison, emotion processing, reward and coping abilities, and interpersonal sensitivity may further contribute but additional studies are warranted.

According to the existing literature, there is a double relationship between mental health disorders and SM addiction risk [59]: pre-existing clinical vulnerabilities may predispose to problematic SM use [60], while problematic SM use may in turn worsen mood, anxiety, and dysregulation via sleep loss, social comparison, and reward–reinforcement mechanisms [61]. This bidirectional association could also be inferred from this study: a significant difference between the clinical sample and healthy controls emerged in both questionnaires, although its interpretation should take into account the modest sample size, the diagnostic heterogeneity of the clinical group, and the fact that none of the healthy controls surpassed either the SOMEDIS or BSMAS cut-off.

According to the latest author knowledge, there is an ongoing debate on the exact contribution of SM addiction to mental health disorders. At the same time, preexisting mental health difficulties can increase SM usage as a coping strategy or a manifestation of these difficulties, and SM addiction can lead to mental health issues. Therefore, a bidirectional relationship exists between SM usage and addiction and youth mental health conditions [59]. In order to more precisely define this association, the authors attempted to investigate whether there was a possible association between a mental health diagnosis and SM addiction risk in the clinical sample.

A direct correlation between emotional dysregulation and problematic internet has been reported in the literature [62,63]. This relationship appears to be mediated by both neurobiological and behavioral factors [64]. Neurobiological factors are known to be related to Internet Gaming Disorder and Internet Addiction (IA); considering Social Media Addiction as a specific area of IA, neurobiological factors could be related to it as well, leading to altered functional connectivity [65] and involving neurotransmitters and neurotrophic factors [66]. Impulsivity and emotion dysregulation appear to be related to addictive behaviors and can be considered as plausible mediators linking SM reinforcement loops with symptom exacerbation [23]. In our sample, a direct relationship was observed between dysregulation patterns and SM addiction risk. It is important to note that a possible limitation is that dysregulation patterns were evaluated based on the clinical diagnosis of emotional dysregulation, cyclothymia, and alimentary dysregulated habits, but no specific questionnaires were used to deeply analyze the clinical profiles. Future research in this direction can be useful to define how dysregulation patterns can have an impact on SM addiction risk and problematic internet use in general considering both the neurobiological and behavioral factors.

An inverse correlation was observed with the diagnosis of tic/obsessive–compulsive disorder and SM addiction risk. While the literature suggests a direct correlation—based on high impulsivity, poor inhibitory control, and technology usage as a coping strategy [7]—our findings may have been influenced by the presence of multiple comorbidities within the sample. In many cases, tic/OCD was not the primary diagnosis, which may have confounded the results.

A modest direct relationship was observed between internalizing symptoms (measured with the CBCL withdrawn depression subscale) and SM addiction risk. This relationship is supported by the existing literature [60]: according to some authors, youth could experience higher levels of social recognition during online interactions, and this can increase the risk of social isolation and time spent online [59]. In addition, a positive association with social anxiety may lead to greater social media usage [67], and social network-addicted users may experience more emotional satisfaction from online activities rather than real life (resulting in a reduced investment in prosocial behavior [68]).

A possible relationship between clinical severity (established with CGI and CGAS scores) and SM addiction was evaluated, but no significant results emerged in the sample. However, it is important to note that in this clinical sample, none of the patients showed SM addiction or problematic internet use as a principal clinical diagnosis.

In our sample, higher SM addiction risk was associated with lower VCI scores on the Weschler Scale. In the current situation, the majority of research projects are focused on analyzing the relationship between social media usage and cognitive abilities such as working memory, problem solving, and attention. In addition, several researchers have focused on possible correlations between language development and screen exposure time in the first years of life, but no clear relationship with verbal cognitive abilities has emerged. Thus, future research on social media usage and verbal cognitive abilities is needed [69].

Empathy is a complex and multifaceted construct that undergoes frequent changes throughout development [10]. Currently, the link between social media use and empathy is still poorly understood, as well as the correlations between empathy and age or gender, and results seem to be inconclusive [15,18]. In our sample, participants with higher SM addiction risk showed lower empathic abilities evaluated through the Perspective Taking and Fantasy IRI subscales; a decrease in the Affective Empathy domain was also observed. At the same time, a direct correlation between the Personal Distress subscale and SM addiction risk emerged. These results are consistent with current literature. Stress appears to be a common vulnerability factor for several addicted-behavior patterns, including SM addiction [70]. The ability of perspective taking and fantasy are important to put oneself in another’s shoes, and this can happen frequently during SM usage. Given this, we could hypothesize that those who experience excessive SM usage could be overwhelmed by the information and connection overload and could obtain the opposite effect, resulting in a decrease in Fantasy and Perspective Taking [19]. In some research, higher levels of affective empathy are found in people with higher social media usage habits [20]. In fact, there are few studies that note that using computers as a gateway to activities such as instant messaging improves what is called “virtual empathy” as well as real-world empathy if it also leads to improved face-to-face communication, in particular with Facebook [71].

To evaluate possible variations based on the social media platform type, we categorized them into four groups: Entertainment, Sharing Content, Profiling, and General Purpose [46]. Significant differences were observed in the empathic abilities measured by the IRI scale. The “Entertainment” category showed higher total scores compared to “Profiling”; similar results emerged for the Fantasy IRI subscale. In the Cognitive Empathy domain, “Profiling” scored significantly lower than both “Sharing Content” and “Entertainment” categories, consistent with the current literature [72]. Since platform categories were derived from a non-validated questionnaire, these results must be considered exploratory. Nevertheless, they may be consistent with the hypothesis that certain online behaviors (i.e., sharing emotions, expressing support) might help to cultivate empathy more than others (i.e., updating profile photos) [20].

Finally, given the rising popularity of short-form-content social media platforms, especially among youth, differences in short-form-content social media use were explored according to clinical diagnosis. A significant difference was observed for ASD: greater use of short-form-content social media was found in participants without ASD diagnosis. This appears in contrast to the common knowledge on repetitiveness in people with ASD but has to be interpreted based on the characteristics of this study. Primarily, as a limitation previously mentioned, participants showed complex and multiple diagnoses with different comorbidities that could have influenced the result. In addition, another potential confounder is that all participants showed high cognitive abilities, and it is known that high-functioning ASD may have different features in the area of repetitiveness. Further research is needed to define a clearer pattern of social media usage and addiction risk that is also based on mental health difficulties.

### Limitations and Future Perspectives

The present study is not without limitations. Firstly, our findings are based on cross-sectional data which makes it impossible to draw conclusions with regard to the causal relationship of the variables included in this study. So further research including experimental and/or longitudinal designs is warranted.

It is evident that the cases and control groups were not perfectly matched with regard to age, with the former being slightly older than the latter, which showed lower variability in the age range. In addition, the study was conducted in few locations of a restricted geographical area, on a population with shared cultural traits. Also because of this, the results here may not be generalizable. The expansion of the sample size is imperative to collect a more varied control group since, for example, none of the actual participants scored clinically in SOMEDIS and BSMAS questionnaires. Thus, creating a greater similarity between the two groups and extending the research to different socio-cultural and economic contexts could facilitate the execution of more significant statistical analysis.

Furthermore, the study was conducted in part through self-report questionnaires and bears the strengths and limitations normally associated with self-report data. As an example, time exposure was estimated through a self-reported frequency; therefore, further studies including objective usage-time parameters (such as time-tracking tools on a single device) can be useful to better define the phenomenon. In addition, longitudinal and content-aware analyses are needed to better define the phenomenon.

Another limitation is the use of a brief, non-validated questionnaire to assess patterns of social media use (e.g., platforms, time spent). While this tool allowed us to capture descriptive information relevant to our aims, the absence of psychometric validation limits the reliability and generalizability of these findings.

The relationship with dysregulation patterns is significant but is only related to a clinical diagnosis. In the future, the development of a more comprehensive definition of the dysregulation pattern, plus incorporating specific questionnaires, has the potential to facilitate a more profound understanding of its correlation with social media addiction risk. Concurrently, given the suggested correlation between social media addiction risk and social anxiety [67], future research could explore this diagnostic feature to enhance our understanding of the relationship between the two conditions.

As technology is constantly changing and evolving, further research can be useful to define a profile of high-risk addiction according to the SM usage patterns in an adolescent sample [2], assessing the differences between ‘digital natives’ and the rest of the population as well as the varying impact of SM on them.

## 5. Conclusions

Social media is deeply integrated into modern society and has rapidly become a prominent feature of daily life, yet its connection with and impact on mental health remains incompletely understood. A precise definition of social media addiction and its inclusion in the diagnostic manuals represents an important challenge for future research. In concordance with the current literature, this study suggest preliminary evidence of associations between SM and mental health of adolescents, including potential effects on empathic and cognitive abilities. Additionally, a relationship was observed between SM addiction risk and dysregulation patterns. Considering that emotional dysregulation is transdiagnostic and continues to emerge in adolescents’ mental health, further research is crucial to better understand its relationship with SM addiction risk. To the best of the authors’ knowledge, only a paucity of studies conducted have taken into regard specific SM platforms and their relationship with addiction risk, empathic abilities, and mental health disorders. Future perspectives should include platform-specific evaluations in larger and longitudinal samples to facilitate a more profound comprehension of the development and chronicity of social media addiction in adolescence. Importantly, the purpose of this research is not to portray specific social media platforms negatively, but rather to explore how distinct usage patterns may relate to vulnerabilities in youth’s empathy and mental health.

## Figures and Tables

**Table 1:** Overview of how each social media platform was assigned to its corresponding category.

Group	Social Media Included
**Entertainment**	TikTok, Twitch, Facebook
**Sharing content**	Instagram, YouTube, Pinterest
**Profiling**	Snapchat
**General Purpose**	WhatsApp

**Table 2:** Overview of clinical diagnoses in the clinical group.

Diagnostic Categories	Number of Patients
**ADHD**	23
**ASD**	4
**Tic Disorder**	3
**Neurodevelopmental Disorder**	18
**Anxiety Disorder**	18
**OCD**	8
**Behavioral Disorder**	65
**Mood Disorder**	4

## Data Availability

The raw data supporting the conclusions of this article will be made available by the authors on request.

## Abbreviations

The following abbreviations are used in this manuscript:

SM	Social Media
SOMEDIS	Social Media Disorder Scale
BSMAS	Bergen Social Media Addiction Scale
IRI	Interpersonal Reactivity Index
RME	Reading the Mind in the Eyes
PSMU	Problematic Social Media Use
AE	Affective Empathy
CE	Cognitive Empathy
DSM-5	Diagnostic Statistical Manual of Mental Disorders—5th Edition
K-SADS-PL	Kiddie Schedule for Affective Disorders and Schizophrenia for School Age Children—Present and Lifetime version
TQI	Total Intelligence Quotient
GAI	General Ability Index
WISC IV	Weschler Intelligent Scale for Children—IV Edition
WAIS IV	Weschler Adult Intelligent Scale—IV Edition
PT	Perspective Taking
F	Fantasy
CE	Empathic Concern
PD	Personal Distress
CGI-S	Clinical Global Impression-Severity Score
CGAS	Children Global Assessment Scales
CBCL	Child Behavior Check List
YSR	Youth Self Report
SD	Standard Deviation
ADHD	Attention Deficit Hyperactivity Disorder
ASD	Autism Spectrum Disorder
OCD	Obsessive–Compulsive Disorder
VCI	Verbal Comprehension Index
SMA	Social Media Addiction
